# Barbed versus conventional 2-layer continuous running sutures for laparoscopic vaginal cuff closure

**DOI:** 10.1097/MD.0000000000004981

**Published:** 2016-09-30

**Authors:** Jin Hwi Kim, Seung Won Byun, Jae Yeon Song, Yeon Hee Kim, Hee Joong Lee, Tae Chul Park, Keun Ho Lee, Soo Young Hur, Jong Sup Park, Sung Jong Lee

**Affiliations:** a Department of Obstetrics and Gynecology, Uijeongbu St. Mary's Hospital; b Department of Obstetrics and Gynecology, Seoul St. Mary's Hospital; c Department of Obstetrics and Gynecology, St. Vincent's Hospital, College of Medicine, The Catholic University of Korea, Seoul, Republic of Korea.

**Keywords:** barbed suture, total laparoscopic hysterectomy, vaginal cuff

## Abstract

We compared results using unidirectional barbed sutures and conventional sutures for vaginal cuff closure during total laparoscopic hysterectomy (TLH).The electronic medical records and surgical videos of 170 patients who underwent TLH between January 2013 and March 2015 at Uijeong-bu St. Mary's Hospital of Catholic University of Korea were reviewed. Vaginal cuffs were closed using the 2-layer continuous running technique with unidirectional barbed sutures (V-Loc; Covidien, Mansfield, MA) in 64 patients and with polycolic acid Vicryl; Ethicon, Somerville, NJ sutures in 106 patients. Procedure time, clinical characteristics, and postoperative complications were compared between the 2 study groups. There were no differences in clinical characteristics (age, body mass index, and demographic data) between groups. The mean suturing time was significantly reduced in the barbed group (7.2 vs 12.2 minutes; *P* < 0.001), although the mean number of stitches was greater than in the Vicryl group (14.1 vs 12.3, *P* < 0.001). Perioperative complications, including episodes of vaginal bleeding, vaginal cuff cellulitis, and postoperative fever, did not differ between groups. There were no instances of vaginal cuff dehiscence in either group. Unidirectional barbed sutures can be used safely to reduce procedure time and surgical difficulty relative to conventional sutures in laparoscopic vaginal cuff closure.

## 1 Introduction

Hysterectomy is one of the most common gynecological surgeries. More than 30,000 hysterectomies are performed in Korea each year. Among these, more than 50% are performed laparoscopically, and this rate is increasing.^[1]^ Laparoscopic hysterectomy has several advantages over traditional open methods such as less pain, less bleeding, shorter hospitalization, and smaller scars. However, successful laparoscopic suturing of the vaginal cuff remains a major hurdle in total laparoscopic hysterectomies (TLHs).^[2]^ As more advanced surgical skills are needed for high-quality laparoscopic suture repair, some studies have reported that the incidence of vaginal cuff dehiscence is higher in laparoscopic hysterectomy than in vaginal or abdominal hysterectomy.^[3–6]^ Although figure-of-eight suturing with intra- or extracorporeal knot tying is commonly used for laparoscopic vaginal cuff suturing due to feasibility, laparoscopic 2-layer continuous running suture is used in our institution, the same as in open abdominal hysterectomy.^[7]^ Therefore, we seek more convenient and safer surgical materials to overcome the learning curve required for laparoscopic suturing.

Barbed sutures have cutting barbs, allowing tensile strength without the need for tying. There is growing evidence that barbed sutures are as safe and well-tolerated as conventional sutures and that their use is associated with reduced operative time in cases of laparoscopic vaginal cuff closure.^[8–12]^ However, most previous studies compared results for continuous running barbed sutures to conventional figure-of-eight sutures, and operative time and procedure time are typically analyzed using medical records rather than surgical videos. Finally, most previous studies involved bidirectional barbed sutures rather than unidirectional sutures.

In this study, we evaluated the efficacy and safety of unidirectional barbed sutures for laparoscopic vaginal cuff closure compared to conventional sutures, all performed by the same 2-layer continuous running technique.

## 2 Materials and methods

Upon Institutional Review Board approval from Uijeong-bu St. Mary's Hospital of the Catholic University of Korea (UC13RISI0102), we carried out a retrospective study of 170 patients who underwent TLH to correct benign conditions between January 2013 and March 2015. From January 2013 to October 2013, 106 vaginal cuff closures were performed with polyglycolic acid sutures (Vicryl; Ethicon, Somerville, NJ). After October 2013, we used unidirectional barbed sutures (V-Loc; Covidien, Mansfield, MA) to close the vaginal cuffs of 64 patients.

All laparoscopic hysterectomies were performed following standard methods using advanced bipolar (EnSeal; Ethicon, Somerville, NJ) and monopolar electrocautery.^[7]^ We used 3 trocars; 1 11-mm trocar for the camera at the umbilicus and 2 5-mm trocars for instrumentation on the lower abdomen. After removal of the uterus, needles were introduced through the umbilical trocar, and the vagina was closed with a 2-layer running suture using Vicryl or V-Loc. Then the needles were removed through the peripheral trocars. In this study, all suturing was performed by a single attending surgeon (JK) who performs over 180 TLHs per year. All laparoscopic surgical procedures were recorded on video. All patients were examined during follow-up visits 1 week, 8 weeks, and 6 months postoperatively. They were advised to avoid intercourse and deep bathing for at least 8 weeks after surgery.

Clinical characteristics including age, body mass index (BMI), medical history, previous surgical history, and pathologic diagnosis were retrieved from electronic medical records. The operative time, time required for vaginal cuff closure, and total numbers of stitches were analyzed by reviewing surgical videos using Pinnacle Studio (Corel, Inc., Ottawa, ON, Canada). The original surgical videos were deleted upon completion of the primary analyses, retaining only anonymized video segments for the secondary analysis. The aforementioned videos were reviewed by a primary reviewer (TCP) and rereviewed by a secondary reviewer (SWB). The reviewers are fellowship-trained practicing gynecologic oncologists (blinded to surgeon identity). Both reviewers independently assessed the videos. In cases of disagreement, the 2 reviewers discussed the videos until consensus was achieved. The operative time was defined as time between insertion and removal of trocars. The time between the beginning of the first stitch and cutting of the last stitch was determined as the suture time.

**Figure 1:**
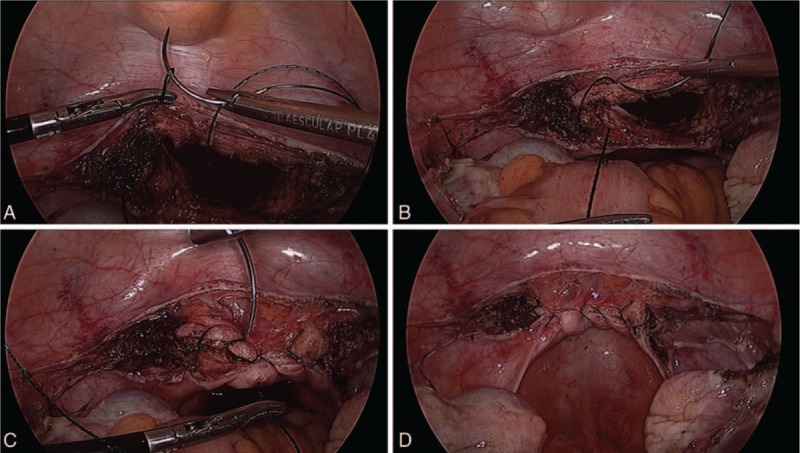
Vaginal cuff closure with unidirectional barbed suture. (A) The repair was started from the left angle passing the needle through the loop of the barbed suture, (B) 6 or 7 stitches placed in a running fashion, (C) then, by continuing backward to the left angle, a second layer was sutured, and (D) 1 or 2 back bites were taken to secure the end of the suture and the suture was cut without remaining from vaginal edge.

For vaginal cuff closures with barbed sutures, we used a 0-polydioxane unidirectional barbed suture on a 37-mm half-circle taper point needle. The repair was started from the left angle of the vaginal cuff to the right angle, with 6 or 7 stitches placed in a running fashion. Then, by continuing backward to the left angle, a second layer was sutured. For barbed sutures, 1 or 2 back bites were taken to secure the end of the suture and the suture was cut without remaining from vaginal edge. Both uterosacral ligaments were incorporated, and the epithelium was also traversed with each bite (Fig. 1).

**Figure 2:**
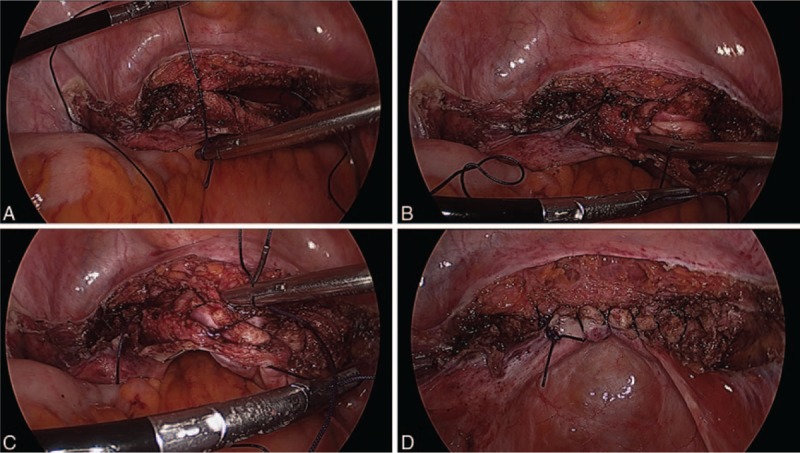
Vaginal cuff closure with Vicryl suture. (A) The repair was started from the left angle using intracorporeal knot tying, (B) repeated tensioning of the suture was required, (C) 2-layer continuous running suture backward to the left angle was placed, (D) the suturing was finished using intracorporeal knot tying.

For Vicryl sutures, we used 1/0 Vicryl suture on a 40-mm half-circle taper point needle that was cut to a 25 cm length for easier handling. A 2-layer continuous running suture was placed, similar to barbed sutures. Because Vicryl sutures cannot maintain tension, intracorporeal knot tying and repeated retensioning of the suture were required, especially using our 3-port laparoscopic system (Fig. 2).

Statistical analyses were carried out using Student *t* test for continuous variables and Fisher exact test for categorical variables. *P* values equal to or less than 0.05 were considered statistically significant. We used Statistical Package for Social Sciences (SPSS) software (version 17.0; SPSS Inc., Chicago, IL) for all statistical analyses.

## 3 Results

V-Loc sutures were used in 64 of 170 operations (37.6%), and Vicryl sutures were used in 106 of 170 operations (62.4%). There were no significant differences in clinical characteristics between groups (Table 1). The mean age in the V-Loc group was 47.6 years versus 46.9 years in the Vicryl group. The mean BMI was 24.1 in the V-Loc group and 24.4 in the Vicryl group.

**Table 1:**
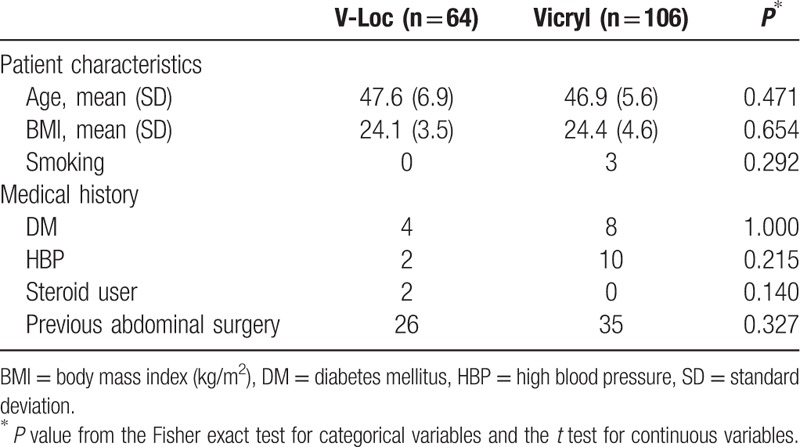
Demographic and clinical characteristics of the 170 patients by suture group.

There were significant differences between groups in vaginal cuff closure time (7.2 ± 1.2 minutes for V-Loc and 12.2 ± 3.3 minutes for Vicryl; *P* < 0.001). The mean number of stitches was greater than in the Vicryl group (14.1 ± 1.5 vs 12.3 ± 1.2; *P* < 0.001). Estimated blood loss, operative time, uterine weight, and duration of hospital stay were comparable between the 2 groups (Table 2).

**Table 2:**
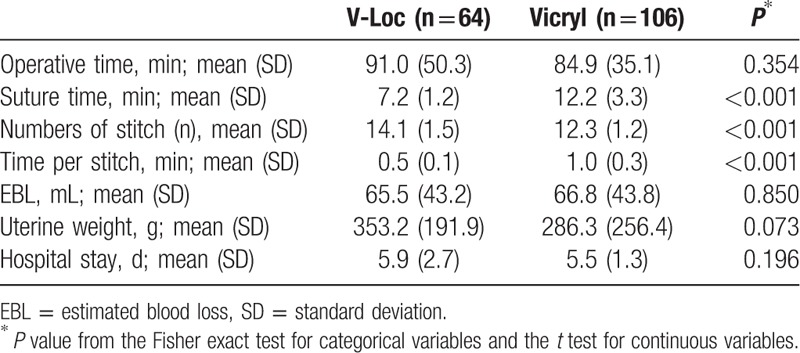
Analysis of surgical outcomes of the 170 patients by suture group.

Postoperative complications in the 2 groups at 1 week, 8 weeks, and 6 months after surgery are shown in Table 3. No differences were observed in bleeding, infection, or postoperative fever between the 2 groups. No vaginal cuff dehiscences occurred in either group, nor did any laparotomy conversions occur in either group.

**Table 3:**
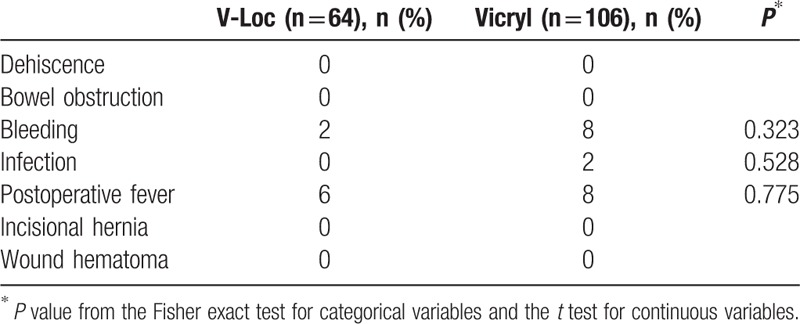
Analysis of postoperative complications.

## 4 Discussion

Hysterectomies can be performed vaginally, abdominally, or with laparoscopic or robotic assistance. Laparoscopic hysterectomies have many benefits including faster return to normal activity, shorter duration of hospital stay, smaller drop in hemoglobin, lower intraoperative blood loss, and fewer wound or abdominal wall infections. However, longer operating times and higher rates of complications, including lower urinary tract injuries and vaginal cuff dehiscence, were found for laparoscopic compared to abdominal hysterectomy.^[3–5]^ These limitations are mainly due to the longer learning curve required for laparoscopic procedures, including laparoscopic closure of the vaginal cuff.^[4,7]^

Although vaginal cuff dehiscence is a very rare complication, estimated to occur at a rate of 0% to 5%,^[3–5]^ it is an adverse event with potential severe morbidity that can cause peritonitis or intestinal evisceration. It is more common after TLH (1.35%) than after total abdominal hysterectomy (0.15%) or total vaginal hysterectomy (0.08%).^[5]^ Excessive electric coagulation at cuff edges and poor quality of laparoscopic suturing are 2 main factors associated with increased risk of dehiscence.^[4,6,13]^

After minimizing electric cauterization, completeness of suturing is very important to reduce such complications. There is growing evidence that 2-layer continuous running sutures are more effective for reducing the incidence of cuff dehiscence than reducing electric cauterization.^[5,6,13,14]^ At our institution, the same 2-layer continuous running suturing method is used in both laparoscopic and open total hysterectomies to achieve the same clinical efficacy.^[7]^ However, laparoscopic continuous suturing with conventional suture materials is a very difficult procedure to master, especially in a 3-port laparoscopic system that has no accessory trocar for the introduction of instruments for maintaining tension.

Currently, 3 types of barbed suture are commercially available: QuillSRS bidirectional barbed suture (Angiotech, Vancouver, BC, Canada), V-Loc unidirectional barbed suture (Covidien, Mansfield, MA), and Stratafix unidirectional and bidirectional barbed suture (Ethicon). These new sutures have barbs that maintain tensile strength evenly along the total length of the wound without knots. Therefore, continuous suturing becomes very simple and maintaining hemostasis is also easy. Theoretically, by using barbed sutures we can reduce the excessive use of electric cautery for hemostasis as well as total procedure time for vaginal cuff closure.

In this study, we observed a significant decrease in time required for vaginal cuff closure with the use of unidirectional barbed sutures compared to conventional sutures. Although our analysis is retrospective setting, this study has some advantages. First, we analyzed surgical videos rather than medical charts and therefore were able to measure suture time very accurately. Second, all of the procedures were performed by a single experienced surgeon who was familiar with both conventional and barbed suture methods. Therefore, we could exclude the influence of skill variability derived from different surgeons. Third, the same 2-layer running suture technique was used in both groups. As a result, we minimized bias and were able to isolate true differences between suture materials used for laparoscopic vaginal cuff closure. Because double-blind settings are impossible to implement in this kind of study, it may enter more bias to the prospective study.

To date, there have been 5 comparative studies of TLH using barbed sutures, except single port and robot-assisted surgery (Table 4). Four studies provided data regarding differences in suturing time between barbed and conventional sutures. In all except 1 study, significant reductions of time required for suturing were observed in the barbed group. Einarsson et al^[11]^ used LapraTy (Ethicon, Inc., Somerville, NJ) clips instead of laparoscopic knot tying in both groups, which might serve to minimize or eliminate differences between groups. Only Bogliolo et al^[10]^ demonstrated a significant difference in total operation times between groups. In myomectomy, in which laparoscopic suturing is the main procedure performed during surgery, the use of barbed sutures significantly reduced operative time.^[15–17]^ Because vaginal cuff closure is not the main procedure performed during TLH and many other factors influence operation time, no differences in total operation time are apparent in studies of TLH including our own.^[8,9,11]^

**Table 4:**
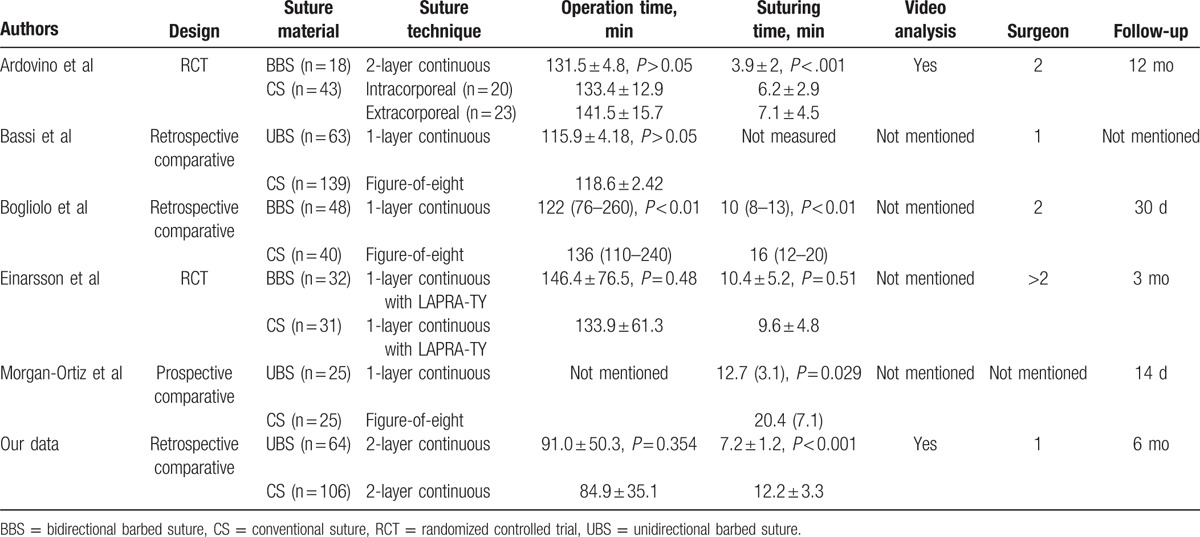
Characteristics and outcomes of comparative studies of TLH with or without barbed suture.

In this study, there were no severe complications such as bowel obstruction or vaginal cuff dehiscence in both groups. In addition, minor complications such as bleeding, infection, and postoperative fever that were able to be recovered by conservative care appeared similar between the 2 groups. Upon early introduction of the use of barbed sutures, small bowel obstruction or volvulus caused by adhesions that the barbs entrapped in the suture may occur.^[18,19]^ However, these problems can be avoided if the remaining is cut shortly from surgical edge. A recent meta-analysis revealed that the use of barbed suture should be considered generally safe and well tolerated.^[20,21]^ Other studies indicate that the use of barbed sutures decreases the incidence of vaginal cuff dehiscence.^[14,22–24]^

In conclusion, we demonstrated that the use of unidirectional barbed 2-layer continuous running sutures for laparoscopic vaginal cuff closure is technically easy and reduces surgical time. Barbed sutures are as safe and well-tolerated as conventional sutures for vaginal cuff closure during TLH. Based on our results and other recent studies, the use of unidirectional barbed sutures is a safe and efficient alternative to conventional sutures for laparoscopic vaginal cuff closure.
